# Editor’s introduction to this issue (G&I 18:1, 2020)

**DOI:** 10.5808/GI.2020.18.1.e1

**Published:** 2020-03-31

**Authors:** Taesung Park

**Affiliations:** Department of Statistics, Seoul National University, Seoul 08826, Korea

In this issue, there are 10 articles: six Original Articles, one Research Communication, two Application Notes, and one article in the category of Clinical Genomics. The first two original articles deal with patient-derived xenografts (PDX). First, Kim et al. (Ewha Womans University, Korea) presented PDX mouse models for 132 lung cancer patients and performed whole-exome sequencing to compare tumor, normal, and xenograft tissues. Through a computational analysis of the somatic mutations and copy number variations, the authors showed that the genomic and histological results agreed well, with more than 90% of concordant cases. Their analyses demonstrate the potential usefulness of PDX mouse models in cancer studies.

Second, Barwe et al. (Alfred I. duPont Hospital for Children, USA) generated 25 primary pediatric acute leukemia samples and their corresponding PDX samples. The authors demonstrated that the primary samples and PDX samples showed a high level of concordance between single nucleotide variants and gene fusions, while other complex structural variants were not as consistent. The high concordance between single nucleotide variants and gene fusions confirms the utility of PDX models for preclinical drug testing.

The third article, by Doulabi et al. (Islamic Azad University, Iran), presents a case-control study of 174 ulcerative colitis biopsy samples and 82 control individuals. The authors performed a candidate gene association analysis of MDM2, which is a phospho-protein and a ubiquitin ligase for p53. The rs309 single nucleotide polymorphism (SNP) detected by the amplification-refractory mutation system PCR technique was shown to be associated with the occurrence of ulcerative colitis. A further study on the direct association of this polymorphism with carcinogenesis is warranted.

Ko et al. (Korea Bioinformation Center, KRIBB, Korea) presented a cloud computing-based system, Bio-Express, for handling a large amount of genomic data. Bio-Express provides user-friendly, cost-effective analysis of massive genomic datasets loaded with multi-omics data analysis pipelines including genome, transcriptome, epigenome, and metagenome pipelines. Bio-Express is a highly efficient cloud computing-based system. Shin at al. (Dankook University, Korea) presented a platform for detection of the Hanwoo-specific structure variation using droplet digital PCR (ddPCR). The ddPCR platform is expected to provide more accurate quantification than PCR and can be applied for the quantitative evaluation of molecular markers.

The final Research Article is by Daoud, who proposed a new robust approach to detecting outliers in a set of segmented genomes of the influenza virus with feature extraction, an alignment-free distance measure, and a mapping into distance space to analyze a quantum of distance values. In his sequel article, the author presents a few technical notes about the distance distribution paradigm used to analyze composite data points in high-dimensional feature spaces. The integrated statistical learning pipeline to process segmented genomes of the influenza virus is illustrated as a sequential-parallel computational pipeline.

In this issue, there are two Application Notes. Park et al. (The Catholic University of Korea, Korea) developed a user-friendly tool, named prediction of avian influenza virus subtype (PAIVS). PAIVS is an analysis pipeline of next-generation sequencing (NGS)-based avian influenza virus (AIV) sequencing data that supports the pre-processing of NGS data, reference-guided AIV subtyping, de novo assembly, variant calling, and identifying the closest full-length sequences by BLAST, and then provides a graphical summary to the end user. Jiang et al. (Seoul National University, Korea) presented the HisCoM-PCA software for performing pathway analysis of SNP data using hierarchical structural component models. HisCoM-PCA is based on principal component analysis (PCA) for the dimensional reduction of SNPs in each gene, and a hierarchical structural component model for pathway analysis. The HisCoM-PCA software has several features. Various selection criteria for the principal component scores in the PCA step can be specified by the user. Multiple public pathway databases and customized pathway information can be used to perform pathway analysis.

The one article in the Clinical Genomics section by Franke and Crowgey (Nemours Alfred I duPont Hospital for Children, USA) provides an evaluation of optimized best practices for genome analysis toolkit (GATK) algorithms, including Parabricks and Sentieon. The evaluation results would be highly informative for users to decide which algorithm of GATK to use to analyze large-scale human genomics datasets.

